# Activation of RhoA,B,C by Yersinia Cytotoxic Necrotizing Factor (CNFy) Induces Apoptosis in LNCaP Prostate Cancer Cells

**DOI:** 10.3390/toxins5112241

**Published:** 2013-11-21

**Authors:** Anke Augspach, Joachim H. List, Philipp Wolf, Heike Bielek, Carsten Schwan, Ursula Elsässer-Beile, Klaus Aktories, Gudula Schmidt

**Affiliations:** 1 Institut für Experimentelle und Klinische Pharmakologie und Toxikologie der Albert-Ludwigs-Universität Freiburg, Albert-Str. 25, Freiburg 79104, Germany; E-Mails: anke.augspach@pharmakol.uni-freiburg.de (A.A.); joachim.list@mars.uni-freiburg.de (J.H.L.); heikebielek@gmx.de (H.B.); carsten.schwan@pharmakol.uni-freiburg.de (C.S.); klaus.aktories@pharmakol.uni-freiburg.de (K.A.); 2 Klinik für Urologie, Universitätsklinikum Freiburg, Engesser Str. 4b, Freiburg 79108, Germany; E-Mails: philipp.wolf@uniklinik-freiburg.de (P.W.); ursula.elsaesser@uniklinik-freiburg.de (U.E.-B.)

**Keywords:** CNFy, apoptosis, prostate cancer, Rho-GTPases

## Abstract

Prostate cancer is the most common malignancy, accounting for about 25% of all incident cases among men in industrialized countries. The human androgen-dependent prostate cancer cell line LNCaP, which is derived from a metastatic lesion of human prostatic adenocarcinoma, is frequently used to study prostate cancer associated signaling pathways *in vitro*. Recently it was described that Rho GTPase activation in these cells leads to apoptotic responses. We used the bacterial toxins CNFy and CNF1, which specifically and directly activate Rho GTPases by deamidation of a single glutamine. We asked whether these Rho activators could induce apoptosis in LNCaP cells. Our results indicate that RhoA activation, induced by CNFy, does lead to intrinsic apoptosis of the cells. Analysis of the underlying signaling pathway reveals that apoptosis induction requires the activity of Rho kinase (ROCK) and myosin activation, an apoptotic pathway previously identified in cancer stem cells.

## 1. Introduction

The bacterial toxins Cytotoxic Necrotizing Factor 1 (CNF1, secreted by pathogenic *Escherichia coli* strains) and Cytotoxic Necrotizing Factor Y (CNFy, produced by *Yersinia pseudotuberculosis*) specifically activate Rho GTPases by deamidation of a single glutamine [1,2]. Both toxins enter a wide spectrum of mammalian cells by receptor-mediated endocytosis [3]. CNF1 activates the Rho GTPases RhoA,B,C, Rac1,2,3 and Cdc42 [4], whereas CNFy selectively activates RhoA,B,C [5,6]. Conflicting reports have emerged regarding the effects of CNF1 on apoptosis. CNF1 reportedly protects cells from apoptosis by Rho-dependent cell spreading and acto-myosin contractility [7]. Moreover, the toxin prevents UV-induced apoptosis of Hep-2 human epithelial cells via activation of Rac and the Akt pathway [8]. In contrast, it has been shown that CNF1 treatment of 5637 bladder cells stimulates an apoptotic response [9]. Nothing is known about the effect of CNFy on apoptosis but since this toxin has more selective substrate specificity when compared to CNF1, the effects on apoptosis are likely to be unique. 

Preventing apoptosis is one of the hallmarks of cancer progression. There is increasing evidence that Rho GTPases play a central role in survival/apoptosis of tumor cells. Moreover, Rho proteins are crucial for other steps of cancer progression, including invasion and metastasis [10,11]. Specifically, activation of RhoA has been linked with induction of apoptosis in tumor stem cells and in prostate cancer [12]. In a recent study, Papadopoulou *et al*. described that activation of Rho GTPases in prostate cancer cells is required for an apoptotic response [13,14]. Xiao *et al*. detected the induction of apoptosis in the prostate cancer cell line LNCaP [15] following phorbolester (PMA) treatment [16]. Interestingly, PMA amongst others leads to activation of RhoA.

Prostate cancer is the most common malignancy with about 25% of all incident cases among men in industrialized countries and represents the second leading cause of cancer deaths [17]. Growth of early prostate cancer is androgen-dependent. Therefore, patients whose tumors are not suitable for surgical intervention or radiotherapy are frequently treated with hormonal intervention to prevent cancer cell growth. However, in most cases androgen-independent tumor cells emerge during the hormonal therapy leading to a clinical relapse and aggressive growth of tumor cells, which are no more accessible for curative treatment [18]. Therefore, it is crucial to understand the signaling pathways that regulate apoptosis in prostate cancer cells. 

Here, we show that activation of RhoA by the specific Rho activator CNFy is sufficient to induce apoptosis in the prostate cancer cell line LNCaP. We also find that in contrast to CNFy, CNF1 treatment results in transient RhoA activation and does not lead to cell death. Further characterization of the underlying signaling pathway reveals that RhoA activation stimulates the intrinsic apoptotic pathway in LNCaP cells. 

## 2. Results and Discussion

Previous studies have determined that activation of RhoA is involved in phorbolester (PMA)-dependent induction of apoptosis in prostate carcinoma cells. We asked whether direct activation of RhoA by the bacterial toxins CNF1 and CNFy is sufficient to induce an apoptotic response in mammalian cells. 

**Figure 1:**
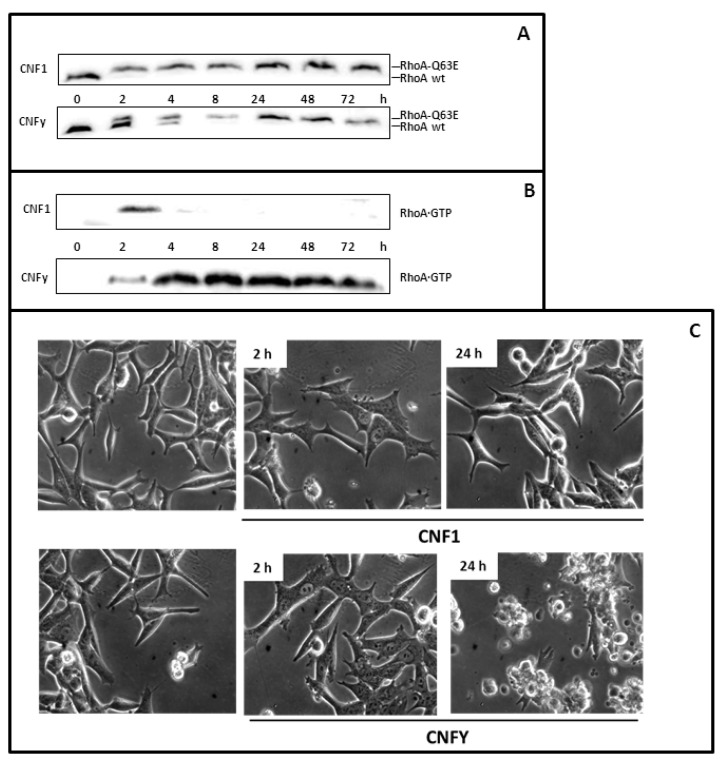
LNCaP cells respond to Cytotoxic Necrotizing Factor 1 (CNF1) and CNFy. (**A**) LNCaP cells were treated for 2 or 24 h with 1 nM CNF1, or CNFy as indicated and lysed. Proteins were separated by urea-SDS-PAGE. RhoA was detected by Western-blotting with a monoclonal anti-human RhoA antibody. Deamidation of RhoA (RhoA-Q63E) was studied by a change in the electrophoretic mobility of the modified GTPase. Note that deamidated RhoA shifts to higher molecular weight; (**B**) The amount of active RhoA was evaluated using pulldown experiments that featured the Rho binding domain of Rhotekin coupled to beads and subsequent Western-blotting with a specific antibody against RhoA (**C**) Morphological changes of untreated and toxin-treated cells were analyzed using an Axiovert 25 microscope (Carl Zeiss). Photographs were taken with a 32× objective. The data shown are representative for more than three independent experiments.

First, we studied whether the toxins are taken up into cells of the prostate carcinoma cell line LNCaP. Deamidation of RhoA by the toxins can be analyzed by SDS-PAGE as modified RhoA exhibits a decrease in electrophoretic mobility relative to unmodified RhoA. We observed modified RhoA in cells treated with CNF1or CNFy (Figure 1A), indicating that both toxins could enter prostate carcinoma cells. Deamidation of RhoA leads to activation of Rho proteins. This can be analyzed by pulldown experiments, using a beads-coupled Rho binding domain of an effector, which exclusively interacts with the GTP-bound form of the GTPase and which is used to specifically enrich active Rho proteins from cell lysates. Our experiments show that CNFy leads to permanent activation of RhoA in LNCaP cells, whereas CNF1 only transiently activates the G-protein (Figure 1B). This observation is consistent with our recent analysis in HEK293 cells [19]. The reason for the transient activation of RhoA is not yet known. Proteasomal degradation of deamidated RhoA following ubiquitination catalyzed by Smurf1 has been shown [20]. However, in our experiments no decrease in the RhoA content of CNF1-treated cells is detectable, suggesting that a different mechanism is involved in negative regulation of RhoA activity.

### 2.1. CNFy Induces Blebbing in LNCaP Cells

**Figure 2:**
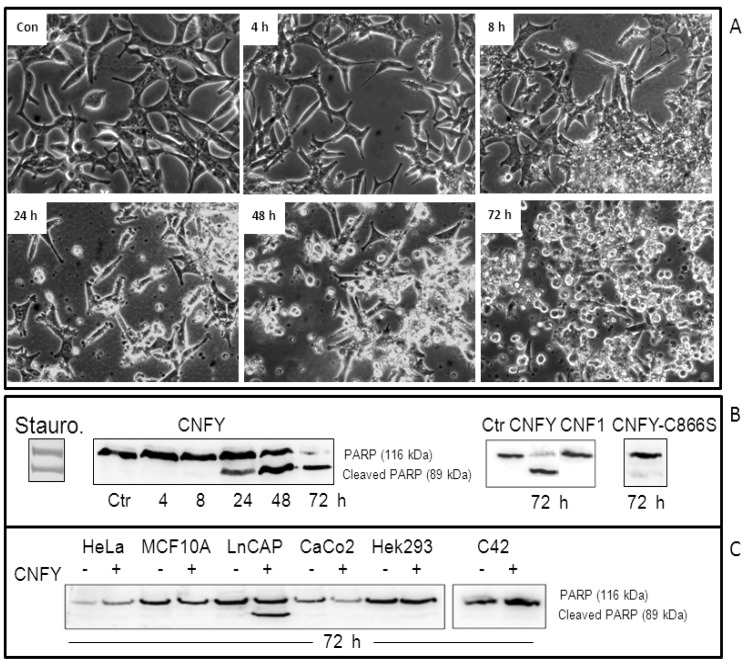
CNFy induces apoptosis in LNCaP cells but not in other cell lines. (**A**) LNCaP cells were treated for up to 72 h with a single dose of CNFy (1 nM). A: Morphological changes were analyzed using an Axiovert 25 microscope (Carl Zeiss) and photographs were taken with a 32× objective; (**B**) Western-blot showing poly (ADP-ribose) polymerase (PARP)-cleavage, which indicates caspase activity. Following toxin treatment, cells were lysed and the lysates analyzed for PARP (116 kDa) and cleaved PARP (89 kDa) by Western-blotting. CNF1 and the catalytically inactive mutant CNFy-C866S did not induce PARP cleavage within 72 h of toxin treatment (right panel), whereas cleaved PARP could be detected 24 h following addition of CNFy or staurosporine (Stauro, left panel) to the culture medium; (**C**) PARP cleavage exclusively occurs in LNCaP cells, but not in CNFy-treated HeLa, MCF10A, Caco2, HEK293 or C42 cells. Data are representative results of more than three independent experiments.

It is well established that treatment of cells with CNFs leads to obvious morphological changes including cell flattening and poly-nucleation [21]. An unexpected phenotype appeared when LNCaP cells were incubated with CNFy. Within 2 h the cells flattened and started to develop bleb-like structures. The toxin-induced changes resulted in cell fractionation detected 24 h after toxin treatment. Cell flattening also occurred with CNF1. However, no blebbing and cell fragmentation could be observed (Figure 1C). To gain more insight into the underlying process of the CNFy-induced death of LNCaP cells, we treated the cells with the toxin and followed the morphological changes by phase contrast and by time-lapse microscopy (Figure 2A). Membrane blebbing could be detected 2 h after toxin addition in a few cells with stronger appearance after 4 to 8 h. Afterwards severe cell fragmentation was detected. However, few intact cells without membrane blebs were still visible. After 5 days, no intact cells could be identified. 

### 2.2. CNFy Induces Apoptosis of LNCaP Cells

**Figure 3:**
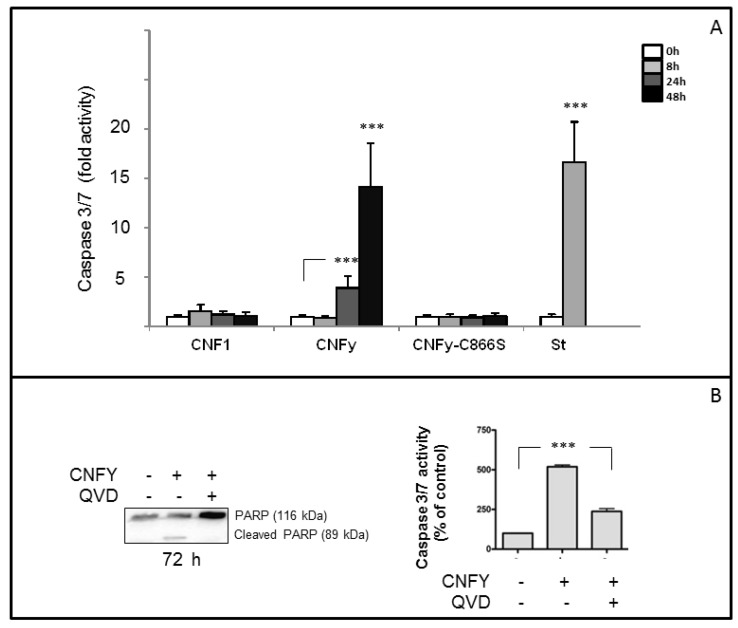
Caspase-3/7 activation in LNCaP cells after incubation with CNFy or CNF1. (**A**) LNCaP cells were incubated with 1nM CNF1 or CNFy as indicated for 0 h (white bar), 8 h (light grey bar), 24 h (grey bar) or 48 h (black bar). Staurosporine (1 µM) was used as positive and the catalytically inactive mutant CNFy-C866S (1 nM) as negative control. Cells were lysed and the activity of caspases 3 and 7 was analyzed by the Apo-One homogeneous caspase-3/7 assay. Shown are data from two independent experiments with four replicates in each case. Given are the mean values with standard deviation; (**B**) PARP-cleavage is blocked in the presence of the caspase inhibitor QVD. LNCaP cells were incubated with toxin for 72 h in the presence and absence of QVD (1 nM). Cleavage of PARP (116 kDa) to an 89 kDa fragment was detected by Western-blotting with an anti-PARP antibody (left panel). Data from three independent experiments were quantified (right panel), (_***_ *p* < 0.001).

Because of the severe blebbing of the LNCaP cells following CNFy exposure, we asked whether the toxin induces apoptosis. Therefore, cleavage of PARP was examined following CNFy intoxication in a time-dependent manner. Cells were treated with the toxin (1 nM) or staurosporine (1 µM) as indicated and checked for PARP-cleavage by Western-blotting (Figure 2B). PARP cleavage increased with time and was nearly complete 72 h following toxin exposure (left panel). PARP-cleavage was neither induced with CNF1 nor with the inactive CNF mutant CNFy-C866S (Figure 2B). Surprisingly, the toxin-induced PARP-cleavage exclusively occurs in LNCaP cells but not in other human cell lines analyzed in our laboratory so far, including cervical cancer cells (HeLa), breast epithelial cells (MCF10A), colon carcinoma cells (Caco-2) embryonic kidney cells (Hek293) and also androgen-independent prostate cancer cells (C42) (Figure 2C). To verify the apoptotic cell response following intoxication with CNFy, additionally caspase-3/7 activation was tested in LNCaP lysates (Figure 3). As negative control, the catalytically inactive mutant of CNFy (CNFy-C866S, 1 nM) was used. Consistent with the data presented above, caspase-3/7 activity in LNCaP cells was induced one day after toxin addition and was increased more than 10-fold after two days (Figure 3A). In contrast, the catalytically inactive mutant of CNFy had no effect on caspase-3/7 activity. Staurosporine (1 µM) induced caspase-3/7 activity more than 15-fold within 8 h. Additionally cleavage of PARP was blocked in the presence of the caspase inhibitor QVD (Figure 3B left panel, quantification: right panel). The data support the notion that CNFy induces apoptosis in LNCaP cells. 

**Figure 4:**
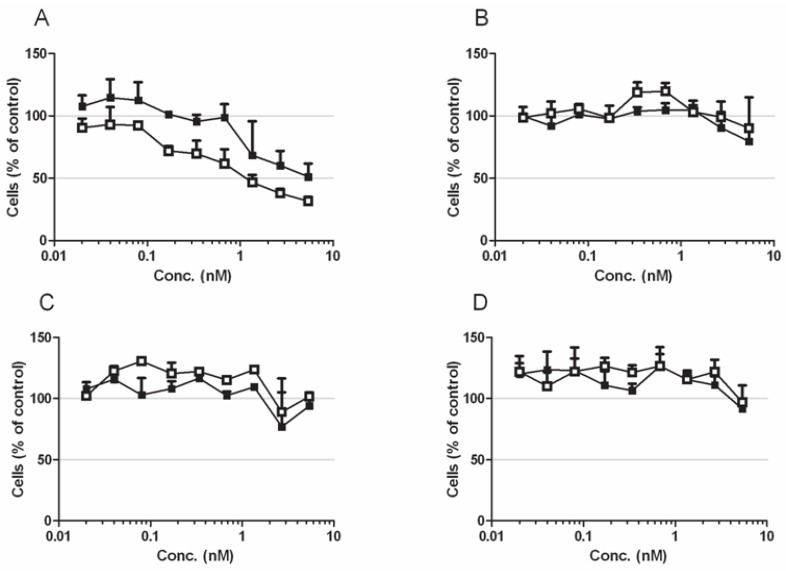
Cytotoxicity of LNCaP and C42 cells. (**A**–**D**) Cytotoxicity of LNCaP (**A**,**C**) and C42 (**B**,**D**) cells, following intoxication with CNFy (**A**,**B**) or CNF1 (**C**,**D**). Cells were incubated with toxin concentrations ranging from 20 pM to 5 nM as indicated for 24 h (black squares) or 48 h (open squares), respectively. Data are presented as percentage of untreated cells. Shown are mean values with standard deviation of three independent experiments each carried out in triplicate.

Next, we asked which concentration of the toxin is sufficient to induce this effect. To this end we investigated the viability of the LNCaP cells following addition of a single dose of CNFy in a quantitative viability assay (WST-1) at 24 h and at 48 h of toxin treatment (Figure 4A). As control we performed the same assay with CNF1, which deamidates Rho, Rac and Cdc42, since this toxin does not induce blebbing and cell fractionation of the cells (Figure 4C). Moreover, the same experiment was performed with the androgen independent prostate cancer cell line C42 (4B-CNFy, 4D-CNF1). As expected, CNFy promoted a concentration and time-dependent reduction in cell viability of LNCaP cells. CNFy concentrations in the picomolar range were sufficient to induce death of LNCaP cells within two days (note that the toxin was applied only once). We determined an EC50-value of about 1 nM CNFy (48 h). On the contrary, there was no decrease in cell viability detectable with CNF1. Moreover, both toxins showed no effect on C42 cells. 

### 2.3. CNFy Induces Plasma Membrane Asymmetry

**Figure 5:**
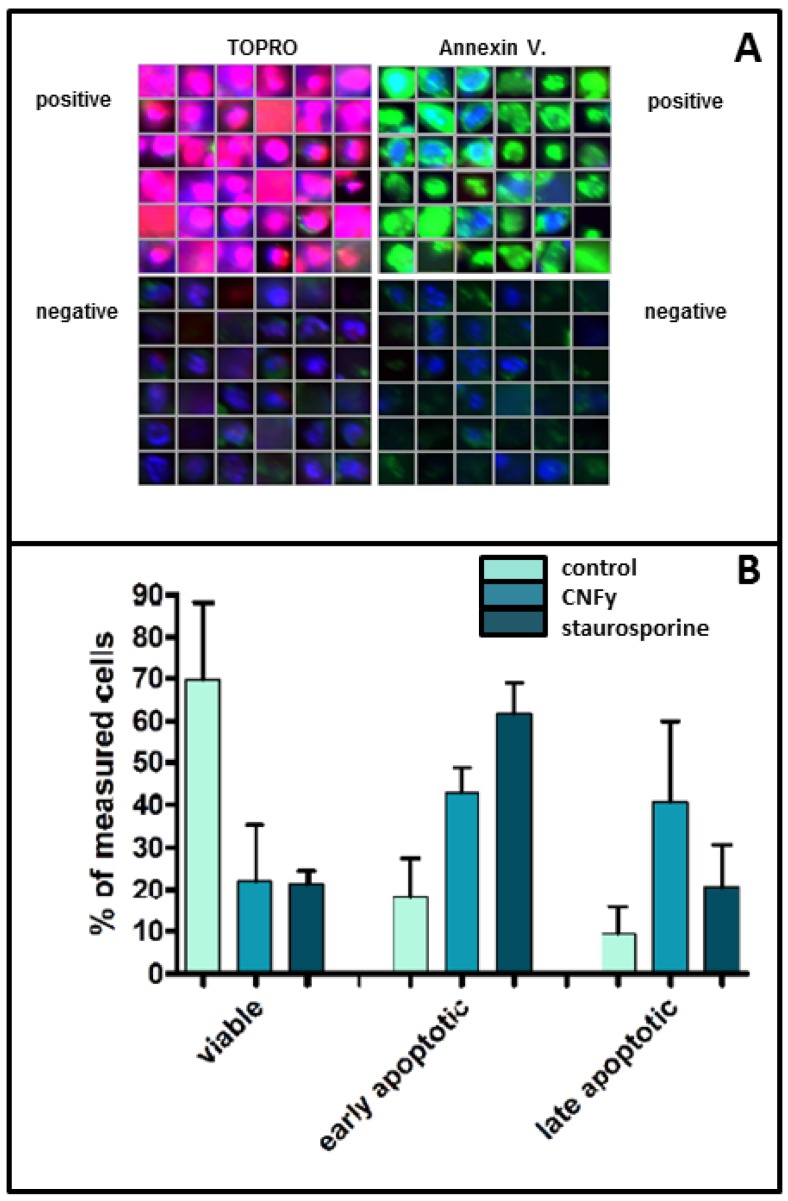
CNFy induces a loss of plasma membrane asymmetry. (**A**) CNFy-treated cells (middle bars) or staurosporine-treated cells (right bars) were stained with PE-labeled annexin 5 (indicating phosphatidylserine on the cell surface) and with Topro (dead cells). Untreated controls are represented in left bars. Cells were analyzed using High Content Screening using an Olympus Scan**^^^**R Screening station (an example of the data originated is shown); (**B**) Viable cells are double negative, early apoptotic are single positive for annexin V alone and necrotic if double positive.

An additional indicator of apoptosis is the loss of plasma membrane asymmetry. To analyze this, we stained toxin-treated cells (middle bars) or staurosporine-treated cells (right bars) with Phycoerythrin (PE)-labeled annexin 5, which binds to phosphatidylserine on the cell surface and indicates the loss of energy-dependent plasma membrane asymmetry. Dead cells were stained using Topro. Cells were analyzed using High Content Screening (an example of the data originated is shown in Figure 5A) and the data were quantified. Results for untreated controls are indicated in left bars. Cells were judged to be viable if double negative, as early apoptotic if positive for annexin V alone and as necrotic or late apoptotic if double positive (Figure 5B). The data show that within 24 h of toxin treatment (1 nM) about 40% of the cells are in an early apoptotic phase and 40% in late apoptosis. This is consistent with the amount of about 60% viable cells detected in the WST assay at this time point (Figure 4A). 

### 2.4. CNFy Induces the Intrinsic Apoptosis Pathway

**Figure 6:**
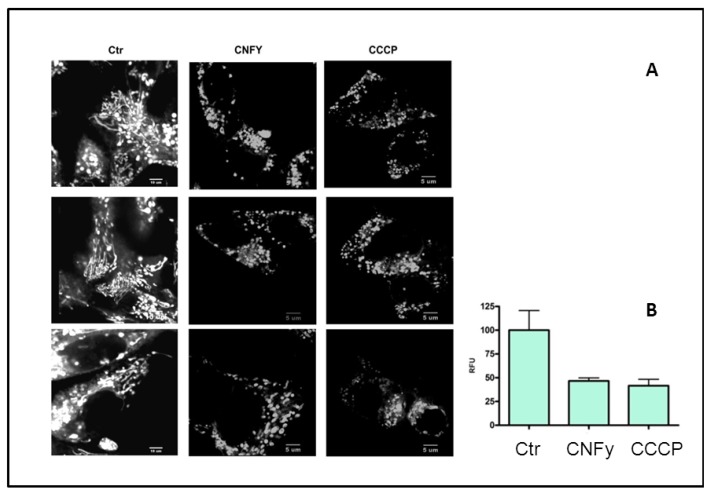
CNFy induces the intrinsic apoptosis pathway. (**A**) To monitor the effect of CNFy on mitochondria, intoxicated cells were stained with MitoTracker Red. CCCP was used as positive control. Mitochondria from CCCP- and CNFy-treated cells were analyzed as tubular or fragmented. Shown are 3 typical examples of cells analyzed; (**B**) For quantitative analysis, cells were stained JC-1. The red/green fluorescence ratio was measured with a fluorescence reader. A decreased ratio indicates mitochondrial depolarization. CNFy induced depolarization of the mitochondria comparable to the CCCP control.

It is well documented that upon initiation of intrinsic apoptosis, mitochondrial depolarization occurs and the tubular networks become fragmented, a process which is governed by the fission machinery. To monitor the effect of CNFy on mitochondria, intoxicated cells were stained with MitoTracker Red and recorded by time lapse microscopy. Carbonyl cyanide m-chlorophenyl hydrazone (CCCP) uncouples the proton gradient during oxidative phosphorylation and was used as positive control. As shown in Figure 6A mitochondria from CCCP- and CNFy-treated cells are fragmented, whereas the tubular network of filamentous mitochondria was visible in the untreated control. For quantitative analysis cells were stained with the membrane permeable dye JC-1. This dye is widely used for determining the mitochondrial membrane potential, because it selectively enters mitochondria and changes color from red to green when the membrane potential increases. Consistent with mitochondrial fission, the toxin induced depolarization of the mitochondria comparable to the CCCP control (Figure 6B).

### 2.5. ROCK and Myosin Activation Is Required for Apoptosis Induction

**Figure 7:**
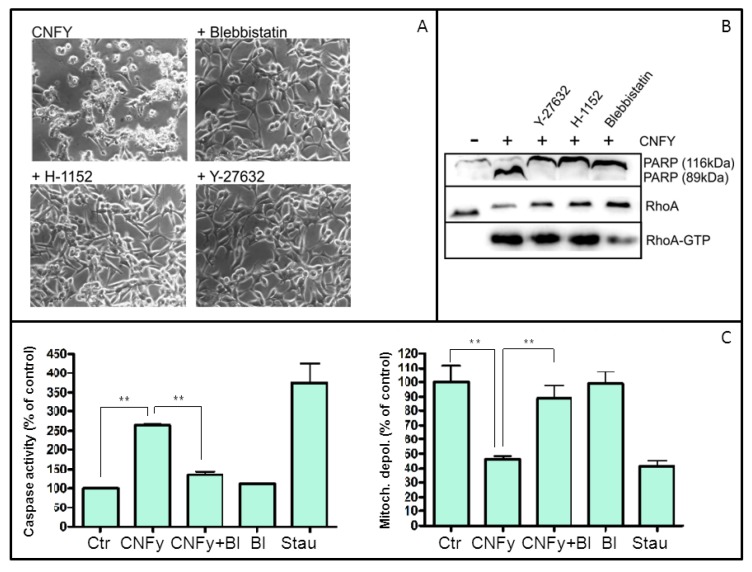
Induction of apoptosis by CNFy requires ROCK and myosin activity. Morphological changes (**A**) and PARP cleavage (**B**) in LNCaP cells after intoxication with CNFy (1 nM) in the presence of two ROCK inhibitors or the myosin inhibitor blebbistatin, respectively, as indicated. Cells were incubated with toxin for 48 h, photographs were taken with a 32× objective and the cells subsequently lysed. Cleavage of PARP (116 kDa) to an 89 kDa fragment was detected by Western-blotting of the lysates with an anti-PARP antibody. Shown are typical results of at least three independent experiments (_**_ *p* < 0.01).

**Figure 8:**
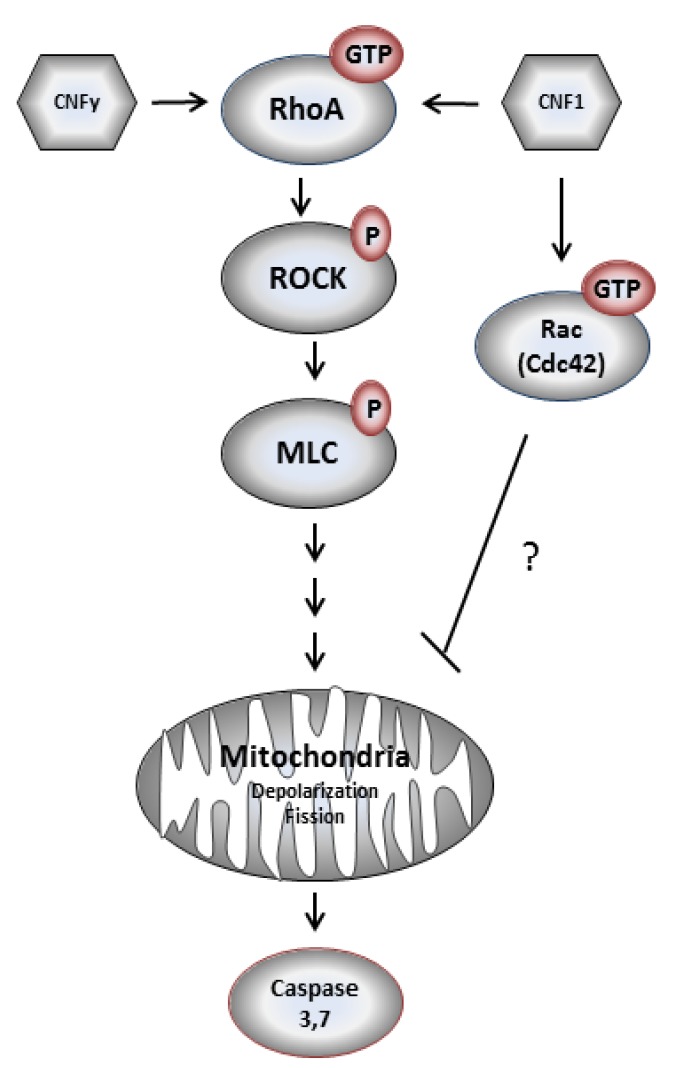
Model of CNFy-induced apoptosis of LNCaP cells.

Recently, it has been shown that Rho-dependent protein kinase (ROCK)-induced hyper-activation of myosin is involved in dissociation-mediated apoptosis of human embryonic stem cells [12]. Therefore, we asked whether this pathway is also required for blebbing and/or apoptosis induction of LNCaP cells. We incubated LNCaP cells with the ROCK inhibitors Y-27632 or H-1152 or with the myosin inhibitor blebbistatin for 2 h and subsequently incubated the cells with CNFy. Induction of blebbing was analyzed by phase contrast microscopy 48 h after toxin addition. As shown in Figure 7A, CNFy was not able to induce blebbing in the presence of either inhibitor. Additionally, cell lysates were checked for PARP cleavage by Western-blotting. As shown in Figure 7B, CNFy-induced PARP cleavage was completely blocked by ROCK or myosin inhibition. In line with this, caspase activity as determined by the Apo one assay (Figure 7C, left panel) and mitochondrial depolarization (Figure 7C, right panel) induced by CNFy were also blocked in the presence of blebbistatin. ROCK and acto-myosin activation are necessary for CNFy-induced apoptosis of LNCaP cells downstream of RhoA. Our findings are summarized in the model of RhoA-induced intrinsic apoptosis depicted in Figure 8.

Our experiments show that CNFy causes a complete and constitutive deamidation of RhoA in LNCaP cells. This persistent activation of RhoA induced by CNFy seems to be sufficient to induce cell death, whereas short term activation of RhoA by CNF1 may not be strong enough to stimulate apoptosis of LNCaP cells. The molecular mechanisms for the transient activation of RhoA by CNF1 are not clear. Doye *et al*. described a transient activation of Rho GTPases in rat bladder cells after CNF1 intoxication, which was based on an increased susceptibility of deamidated Rho GTPases to ubiquitination and proteasomal degradation [22]. On the other hand, Sander *et al*. found that Rac1 signaling was able to antagonize RhoA activity by cross-talk [23]. Therefore, the broader substrate specificity of CNF1, including deamidation of Rac may be the cause for LNCaP survival. This hypothesis is reinforced by studies with human epidermoid cancer cells (HEp-2), in which CNF1 caused Rho activation with subsequent actin filament reorganization, cell spreading and multi-nucleation. Rac and Rho activation led to an up-regulation of the anti-apoptotic proteins Bcl-2 and Bcl-xl and therefore promotion of cell survival [7].

**Figure 9:**
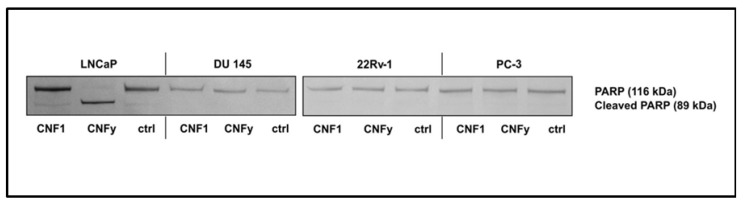
PARP cleavage in prostate cancer cell lines by CNFs. LNCaP, DU 145, 22Rv-1 and PC-3 cells were incubated with CNF1 or CNFy (1 nM each) for 72 h. PARP cleavage was detected by Western-blotting with an anti-PARP antibody. Shown are typical results of at least three independent experiments.

The reason for the transient activation of RhoA by CNF1 and the pathway blocking CNFy-induced apoptosis of LNCaP cells will be analyzed in future studies. Interestingly, within the group of analyzed prostate carcinoma cells exclusively LNCaP cells, which are the only androgen dependent cells, responded with apoptosis induction upon CNFy-dependent Rho activation. Three additional prostate carcinoma cells (DU 145, 22Rv-1 and PC-3) were tested. CNFy was not sufficient to induce apoptosis in these cells, as detected by morphological changes and PARP cleavage (Figure 9). In contrast to LNCaP cells, these cell lines are all androgen insensitive. It will be interesting to analyze whether this property of the cells is the reason for the missing apoptotic response. All cell lines analyzed showed similar kinetics of RhoA deamidation and activation by both toxins and therefore, no difference in the uptake of CNFs. However, we detected more active Rac in the untreated androgen insensitive cell lines (data not shown), suggesting that active Rac may counteract the RhoA-induced apoptosis (compare model in Figure 8).

In view of all these studies, CNFy may be considered as a candidate for the treatment of prostate cancer. The bacterial toxins *Pseudomonas aeruginosa* Exotoxin A and Diphtheria toxin from *Corynebacterium diphtheriae* are prominent examples for the construction of immunotoxins, in which the catalytic part of the toxins were coupled to tumor-specific antibodies or ligands for the targeted treatment of different cancers (rev. in [24]). The use of CNFy-based immunotoxins directed against prostate tumor cells could therefore represent a future alternative for hormone deprivation therapy avoiding selective pressure, which leads to hormone-independent cancer growth.

## 3. Experimental Section

### 3.1. Cell Lines

Human prostate cancer cell lines LNCaP, C4-2, DU 145, 22Rv-1 and PC3 were grown in RPMI 1640 medium (Invitrogen, Carlsbad, CA, USA) with 15% FCS in the presence of penicillin (100 U/mL) and streptomycin (100 mg/L) at 37 °C in a humidified atmosphere of 5% CO_2_. HeLa, Hek293 and CaCo2 cells were grown in Dulbecco’s modified Eagle’s medium (DMEM) supplemented with 10% fetal calf serum, 1% nonessential amino acids, penicillin (4 mM), and streptomycin (4 mM). For inhibition of Rho kinase, Y-27632 (Biozol, Eching, Germany) or H1152 (Tocris, St. Louis, MO, USA) was used as indicated. The myosin inhibitor Blebbistatin was obtained from Sigma, St. Louis, MO, USA. 

### 3.2. Preparation of Recombinant Protein Toxins

For toxin purification, BL21 *E*. *coli* strains, carrying pGEX-CNF1, pGEX-CNFy or the plasmids with the catalytically inactive mutants pGEX-CNF1-C866S or pGEX-CNFy-C866S were grown in LB-medium, respectively. At OD 0.6, protein synthesis was induced by the addition of 0.1 mM IPTG. Following further incubation for 2 to 4 h, cells were collected by centrifugation and lysed by sonication in lysis buffer (20 mM Tris-HCl (pH 7.4), 10 mM NaCl, 1% Triton, 1 mM phenylmethylsulfonylfluoride (PMSF) and 5 mM dithiothreitol (DTT)). The toxins were purified as GST-fusion proteins by affinity chromatography with glutathione-sepharose (GE Healthcare, Freiburg, Germany). Loaded beads were washed five times with lysis buffer (without PMSF) at 4 °C. The GST-CNF fusion proteins were eluted from the beads by glutathione (10 mM glutathione and 50 mM Tris-HCl (pH 7.4)) twice for 10 min at room temperature.

### 3.3. Cell Morphology

For analysis of morphological changes induced by intoxication, LNCaP cells were treated for several time intervals with 1 nM CNFy, CNF1 or the respective inactive mutants. Morphological changes were analyzed using an Axiovert 25 microscope (Carl Zeiss, Jena, Germany). 

### 3.4. Biochemical Analysis of Rho GTPase Deamidation

Cells were seeded on 10 cm cell culture dishes, grown over night, intoxicated with CNFs for 2 to 72 h, washed with PBS and lysed in 250 µL lysis buffer, containing 50 mM Tris-HCl (pH 7.4), 100 mM NaCl, 2 mM MgCl_2_, 10% (*v/v*) glycerol and 1% (*v/v*) Igepal. Then cells were scraped off and the lysate was centrifuged for 30 min at 14.000 rpm at 4 °C. 50 µg of lysate per lane was separated on urea containing SDS-gel. After blotting onto PVDF membranes, blots were developed using mouse anti-RhoA IgG (Santa Cruz Biotech, Santa Cruz, CA, USA) as primary and goat anti-mouse IgG-HRP as secondary antibody (Upstate, Lake Placid, NY, USA). Finally, blots were developed using enhanced chemiluminescence. 

### 3.5. Pull down Experiments

The RhoA-binding region (RBD), encoding the *N*-terminal 90 amino acids of Rhotekin (Rhotekin pull-down) or the Cdc42- and Rac-binding CRIB domain (amino acids 56–272) of PAK (PAK pull-down) were expressed as GST-fusion proteins in *E*. *coli* BL21. Cells were lysed in Rhotekin lysis buffer (50 mM Tris-HCl (pH 7.5), 150 mM NaCl, 5 mM MgCl_2_, 10% (*v/v*) glycerol, 0.5% (*v/v*) Triton X-100 and 1 mM DTT). The GST-coupled Rhotekin-RBD or PAK-CRIB was purified by affinity chromatography with glutathione-Sepharose. Loaded beads were washed three times with Rhotekin lysis buffer and once with buffer A (10% glycerol, 50 mM Tris-HCl (pH 7.4), 100 mM NaCl, 1% NP-40, 2 mM MgCl_2_ and 0.5 mM PMSF).

Toxin-treated or control cells were lysed in buffer A and the lysates were cleared by centrifugation. A fraction of cleared lysates (50 µg of total protein) was analyzed by Western-blotting (input). 1 mg of total lysate was incubated with protein-loaded beads for 1 h at 4 °C by head-over-head rotation. After incubation, beads were washed once with buffer A. Samples were boiled in Laemmli buffer and separated by SDS-PAGE. RhoA and Rac1 were analyzed by Western-blotting with their specific antibodies. 

### 3.6. Cytotoxicity

Cytotoxicity of the CNF toxins to LNCaP cells was determined using the WST-1 viability assay. The absorbance of the formazan dye can be quantified spectrophotometrically and directly correlates with the cell number.

For WST-1 assays, cells were seeded in a 96-well plate (1.5 × 10^4^ cells per well) and grown for 24 h. Then the toxins were added at concentrations between 0 and 5.4 nM to the cells followed by incubation for 24 or 48 h. Then WST-1 reaction solution was added and the absorbance of the samples measured at 450 nm (ref. 690 nm). Cytotoxicity was defined as the toxin effective concentration, inducing a 50% reduction in cell viability relative to untreated control cells (EC50-value).

### 3.7. Apoptosis

Induction of apoptosis can be measured by analyzing the amount of active caspases in cell lysate. To assess active caspase levels we used the “ApoOne Homogeneous Caspase-3/7 Assay” (Promega, Mannheim, Germany). The cleavage-derived fluorescence is proportional to the amount of active caspases 3 and 7. The assay was performed following the manufacturer´s instructions. Shortly, LNCaP cells were seeded in 96-well-plates, cultivated overnight and treated with the toxins as indicated. The apoptosis-inducing reagent staurosporine (1 µM) was used as positive control and the catalytically inactive CNF mutant CNFy-C866S as negative control. After toxin incubation, the Apo-ONE-Caspase-3/7-reagent was added and the plates were incubated in the dark. Fluorescence was measured in a multi-well plate reader at 499 nm (emission 521 nm). 

Poly (ADP-ribose) polymerase (PARP) cleavage by caspase-3 is a hallmark of apoptosis. For the measurement of PARP cleavage, cells were seeded in 6 well plates and intoxicated with 1 nM CNFy or CNF1 for different time periods. Inactive toxin mutants were used as negative controls. After washing with ice-cold PBS, cells were incubated with 50 mM Tris-HCl (pH 7.4), 100 mM NaCl, 2 mM MgCl_2_, 10% (*v/v*) glycerol, 1% (*v/v*) Igepal and 4% (*v/v*) protease inhibitor solution (Roche Diagnostics, 1 tablet dissolved in 2 mL water) for 5 min, scraped and centrifuged for 30 min at 14,000 rpm at 4 °C. Cell lysates (50 µg) were separated by SDS-PAGE and transferred onto PVDF membranes.

After blocking with 5% (*w/v*) non-fat dried milk in TTBS for 60 min, membranes were incubated with rabbit anti-PARP IgG (New England Biolabs, Ipswich, MA, USA) at 4 °C overnight and washed. Goat anti-rabbit-HRP (Biotrend, Köln, Germany) was used as secondary antibody (30 min, room temperature). Finally, blots were developed using enhanced chemiluminescence. The caspase inhibitor QVD was used in a concentration of 1 µM.

### 3.8. Measurement of Plasma Asymmetry

Cells were plated into an 8 well µ-slide (Ibidi, Martinsried, Germany) and treated with CNFy (1 nM) or staurosporine (10 µM) at 37 °C, overnight. Cells were stained with Annexin V conjugated to Phycoerythrin (PE) for 15 min at 4 °C in the dark. Just prior to analysis, TOPRO 3 Iodid was added at a final dilution of 1:10,000 into each sample. Cells were analyzed using High Content Screening with an Olympus Scan**^**R Screening station. Cells were judged to be viable, if double negative, as early apoptotic, if positive for Annexin V alone and as necrotic or late apoptotic, if double positive. Data were quantified from 3 independent experiments, including at least 36 image positions analyzed in each scan.

### 3.9. Mitochondrial Fission and Depolarization

Cells were seeded in glass bottom dishes (Cell view glass bottom dishes) and the next day intoxicated with CNFY or carbonyl cyanide m-chlorophenyl hydrazone (CCCP) as positive control. For mitochondrial observation cells were treated with MitoTracker Red (100 nM) 30 min prior to time lapse microscopy. The culture medium was exchanged and the cells recorded for 4 h under 40× magnification. For quantitative analysis, cells were seeded into a 96 well plate and treated with CNFy or CCCP. They were then stained with JC-1 (5 µg/mL). Mitochondrial membrane depolarization causes a shift in emitted light from 530 nm to 590 nm when excited at 488 nm. Suspension was incubated for 30 min at room temperature and the emission was measured with an Infinite M200 fluorescence reader. A decrease in the red/green fluorescence intensity ratio equals mitochondrial depolarization. Data were quantified for at least 3 wells each of 3 independent experiments.

### 3.10. Statistical Analysis was Performed Using One Way ANOVA (Bonferroni)

Videos: Movie of DIC time-lapse microscopy of LNCaP cells after CNFy treatment (1 nM, V 1) or of untreated control cells (V 2). Sub-confluent LNCaP cells were treated with 1 nM CNFy. Pictures were taken every 90 s for 6 h by a CCD-camera (Coolsnap HQ; Roper Scientific, Tucson, AZ, USA) driven by Metamorph imaging software (Universal Imaging, Downingtown, PA, USA). Cells were incubated at 37 °C in a heated chamber that provided a humidified atmosphere (6.5% CO2 and 9% O2) on a Zeiss Axiovert 200 M inverted microscope (Carl Zeiss GmbH, Jena, Germany) with a 40× Plan-Apochromat objective. Scale bar represents 20 µm.

## 4. Conclusions

We show with several methods that strong RhoA activation induced by the bacterial toxin CNFy leads to intrinsic apoptosis in the androgen-dependent prostate cancer cell line LNCaP. This is surprising, because activation or overexpression of Rho GTPases is known to promote tumor-genesis in most cancer entities. Apoptosis induction in LNCaP cells requires the activity of Rho kinase (ROCK) and myosin. This finding is important, because ROCK inhibitors are discussed to be useful agents for cancer treatment. 
